# Can Corticospinal Excitability Shed Light Into the Effects of Handedness on Motor Performance?

**DOI:** 10.3389/fnrgo.2021.651501

**Published:** 2021-03-19

**Authors:** Marco Antonio Cavalcanti Garcia, Anaelli Aparecida Nogueira-Campos, Victor Hugo Moraes, Victor Hugo Souza

**Affiliations:** ^1^Programa de Pós-Graduação em Ciências da Reabilitação e Desempenho Físico-Funcional, Faculdade de Fisioterapia, Universidade Federal de Juiz de Fora, Juiz de Fora, Brazil; ^2^Laboratório de Neurofisiologia Cognitiva (LabNeuro), Departamento de Fisiologia, Instituto de Ciências Biológicas, Universidade Federal de Juiz de Fora, Juiz de Fora, Brazil; ^3^Grupo de Estudos em Neuro Biomecânica, Faculdade de Fisioterapia, Universidade Federal de Juiz de Fora, Juiz de Fora, Brazil; ^4^Núcleo de Pesquisas em Neurociências e Reabilitação Motora, Universidade Federal do Rio de Janeiro, Rio de Janeiro, Brazil; ^5^Instituto de Biofísica Carlos Chagas Filho, Universidade Federal do Rio de Janeiro, Rio de Janeiro, Brazil; ^6^Department of Neuroscience and Biomedical Engineering, Aalto University School of Science, Espoo, Finland

**Keywords:** motor evoked potential, transcranial magnetic stimulation, lateral preference, handedness, motor skill

## Introduction

Handedness is characterized as a lateral preference or advantage for one side of the body over the other when performing sensory or motor tasks (Cochet and Byrne, 2013). Different hypotheses have been formulated to explain this enigmatic human feature (Papadatou-Pastou et al., 2020). Genetic background (Annett, 1978; Paracchini and Scerri, 2017) anatomical or physiological asymmetries throughout the nervous system (NS) development or in adult life may contribute to the framework of lateral preference in primates (Daligadu et al., 2013). Understanding the origin of such asymmetries is crucial for the development of efficient patient-specific rehabilitation and training programs. Previous studies using neuroimaging (Amunts et al., 2000; Hervé et al., 2005) and neurophysiological techniques (Hammond et al., 2004; Souza et al., 2018) sought to quantify peripheral and central NS anatomical asymmetries using cadavers, for instance (White et al., 1994).

Alternatively, Transcranial Magnetic Stimulation (TMS) is a powerful tool to probe the brain function non-invasively *in vivo*. It has been used to unveil how the central NS drives the lateralization of motor gestures under different body postures on active and resting conditions (Triggs et al., 1994; Brouwer et al., 2001). TMS elicits motor evoked potentials (MEP) in a target muscle when delivered over the primary motor cortex (M1). Thus, monitoring changes in the MEP parameters provides a measure of corticospinal excitability (CSE) and relevant information about the integrity of the corticospinal tract (CST), especially in clinical conditions (Rossini et al., 2015). In the following topics, we describe recent findings and suitable recommendations for a more in-depth analysis of how CSE has been used for evaluating neurophysiologic attributes of handedness.

## Anatomical Features Underlying the CSE and Handedness

According to White et al. (1994), “*humans have more cortical (and presumably subcortical) circuitry devoted to the representation of the right upper extremity than to the left*.” Thus, one might hypothesize that the CST would be larger on the right side of the spinal cord for right-handers. Likewise, the contrary stands true for left-handers even though the low prevalence limits the cadaveric approaches (9.3–18.1%, Peters et al., 2006; Papadatou-Pastou et al., 2020). However, the reported results are inconclusive due to the limited number of studies conducted on cadavers. For instance, Yakovlev and Rakic (1966) and Melsbach et al. (1996) reported larger tracts in the right side of spinal cord of neonates and adult specimens. In contrast, Kertesz and Geschwind (1971) and White et al. (1997) did not observe any difference between both sides of medullae in 158 and 67 adult specimens, respectively. If the dominant side exhibits a larger CST, one might hypothesize that single-pulse TMS would recruit larger neuronal populations and, therefore, lead to higher MEP amplitudes in the dominant than the contralateral side. Indeed, few studies observed different MEP responses between dominant and non-dominant cerebral hemispheres (Triggs et al., 1994; Matsunaga et al., 1998).

Interestingly, asymmetries in the CST in neuroimaging studies (Ciccarelli et al., 2003; Westerhausen et al., 2007) or MEP amplitudes (Garcia et al., 2020) between both sides of the human body have been refuted. Alternatively, neuroimaging studies support the hypothesis that asymmetries in the right- and left-handers take place at the cellular microstructure level throughout different NS regions, including the CST (Andersen and Siebner, 2018). Then humans are provided with more complex cortical and subcortical circuits devoted to benefit one side in specific motor tasks (Jang et al., 2017). Li et al. (2014) also suggest that handedness manifests in local neural networks due to “*high local clustering and short paths between nodes*” that can contribute to more significant asymmetries for right- but not left-handers. Their findings are partially supported by Hervé et al. (2005). They observed a positive correlation between contralateral gray matter volume and hand skills. It could be due to more complex neural networks with greater functional capacity. We might speculate that asymmetries in the CSE between the dominant and non-dominant sides observed in precision tasks (Triggs et al., 1994) were due to the extent of neural networks recruitment.

Consequently, CSE might better assess the degree of handedness when subjects are performing fine motor tasks, suggesting that some singularities of “dominant” neural networks recruited may be uncovered. Moreover, we should note that the TMS pulse recruits polysynaptic circuits mediated by inhibitory and excitatory interneurons and pyramidal neurons projecting into the CST (Di Lazzaro et al., 2018). Thus, one can argue that possible differences in MEP parameters from the dominant and non-dominant sides reside in the complexity of these high-order circuits engaged in performing motor tasks that do not require efforts above ~10% of the maximal strength (Triggs et al., 1994) when compared the motor thresholds of different muscles from both upper limbs in the right- and left-handers. It was conducted during writing and under rest conditions. It is also suggested that motor tasks that require contractions above the mentioned level may normalize the cortical excitability and consequently mask differences in CSE (van de Ruit and Grey, 2016).

## Methodological Approaches on MEP Recording Applied to Handedness

Three distinct MEP parameters are widely used to evaluate the CSE: latency, resting motor threshold (rMT) and peak-to-peak amplitude. Latency defines the onset time of the myoelectric activity evoked by the TMS pulse and may indicate pathological conditions such as demyelination (Fernández et al., 2013). Latency depends on the conduction velocity of the neural drive in the CST (Kidgell and Pearce, 2011). In this case, possible anatomical asymmetries associated with the ratio between fast and slow fibers would lead to distinct latencies from the dominant and non-dominant sides. Then, one may conjecture that the size principle could explain the differences in motor nerve latencies between both sides (Henneman et al., 1965). It is based on the concept that motor units containing axons with smaller diameters would have a lower conduction velocity and longer latencies. Curiously, most TMS studies did not report significant differences in MEP latencies in rest conditions between dominant and non-dominant sides (Kallioniemi et al., 2015), suggesting that latency in such condition may not be affected by manual dominance. Latency is frequently calculated by visual inspection and manually annotated in the surface electromyography (sEMG) recording. The manual annotation exhibits a high intrarater reliability (Livingston and Ingersoll, 2008) and the visual definition of onset time is subjective (Brown et al., 2017). Therefore, one should carefully interpret the physiological outcomes based on manually annotated latencies. An automated method was recently proposed by Šoda et al. (2020) and might be a better candidate to detect differences between dominant and non-dominant sides.

Likewise, asymmetries in the rMT have been used to understand how handedness manifests itself. The rMT represents the minimal TMS intensity delivered to M1 to elicit MEPs higher than a defined amplitude, e.g., 50 μV (Rossini et al., 2015). Macdonell et al. (1991) reported lower rMT for the dominant cerebral hemispheres of right-handers. In contrast, Davidson and Tremblay (2013) recorded higher rMT in the dominant hemisphere of left-handed individuals. Remarkably, many other studies did not observe any significant rMT difference between both cerebral hemispheres (Livingston et al., 2010; Cueva et al., 2016; Garcia et al., 2020), refuting the previous findings. Even though Brouwer et al. (2001) also reported similar rMT between hands in right and left-handers, MEPs were higher when stronger hand muscles were recruited. It was hypothetically associated with the dominant side. Their findings also reinforce that MEP parameters related to the degree of handedness might be accessed when performing a motor task, as previously hypothesized.

Interestingly, a few authors reported other variables such as gender and age as possible intervening factors in handedness (Sala et al., 2017). In this respect, Amunts et al. (2000) reported anatomical asymmetries between cerebral hemispheres for right-handed males, but not females. In turn, Livingston et al. (2010) did not observe differences in MEP parameters when comparing both genders and right- and left-handers. According to Matsunaga et al. (1998), MEP parameters seem to be even less pronounced in older than young people.

MEP amplitude is associated with the level of motor unit recruitment. It may be affected by several factors such as postural adjustments (Sasaki et al., 2018), the type of task (rest *vs*. active) (Semmler and Nordstrom, 1998; Brouwer et al., 2001), and muscle length (Chye et al., 2010). Thus, methodological issues such as surface electrodes placement and the type of task performed, e.g., rest *vs*. active, may explain conflicting findings on CSE asymmetries between dominant and non-dominant sides (Triggs et al., 1994). For instance, Daligadu et al. (2013) observed an asymmetrical pattern on the stimulus-response curve between the dominant and non-dominant cerebral hemispheres when TMS intensity was 90–150% of rMT. Interestingly, they found a lower rMT for the non-dominant side in right and left-handers, mainly for TMS intensities above 120% of rMT. This observation contrasts to the conventional idea that the dominant hand presents a lower level of excitability. The underlying mechanisms related to handedness under resting or active conditions may pose different views from the CSE and its anatomical and neurophysiological substrates. Souza et al. (2018) observed differences in MEP spatial distributions when using high-density sEMG, which were significantly shifted toward the lateral border of the thenar region on the dominant hand and might offer some advantage in generating torque in the metacarpal phalangeal joint. They suggest a biomechanical background on handedness according to the MEP spatial distribution and not solely on the amplitude. Therefore, alternative methodological approaches might provide additional insights into the effect of manual dominance on the CSE measures.

## The Measurement of the Degree of Handedness

Questionnaires have been widely used to evaluate the degree of handedness (Oldfield, 1971). For instance, the Edinburgh Handedness Inventory (Oldfield, 1971) estimates the manual preference by a laterality quotient (LQ). Davidson and Tremblay (2013) observed that the LQ correlates with the degree of manual dexterity. Manual dexterity may also be associated with MEP parameters (Souza et al., 2018), which is postulated as derived from the degree of handedness. Nonetheless, there have been various adaptations on the original questionnaire (Edlin et al., 2015), which might have negatively impacted the agreement between different studies. Thus, the lack of uniformity or consensus in the use of Oldfield's and other questionnaires to determine the degree of laterality entangles the standardization of measurements and, consequently, the comparison between multiple studies. Flindall and Gonzalez (2019) go further on these issues, suggesting that many participants may report differences in the preferred hand for a task when asked on separate days for only a few weeks. Therefore, active motor tasks to quantify the degree of handedness seem to be a viable alternative (Cavill and Bryden, 2003; Flindall and Gonzalez, 2019). Indeed, a task-oriented evaluation may contribute to obtaining more clear discrimination of the degree of handedness, besides being correlated with anatomical and neurophysiology mechanisms. Registering the MEP while performing the motor tasks addressed by these questionnaires could contribute to evaluating the hypothetical relationship between the observed scores and the corticomotor pathway excitability.

## Conclusion

The CSE might reveal underlying mechanisms that contribute to assess the degree of handedness, especially in active tasks. Moreover, MEPs might decode the central NS effort to drive a motor task, including biomechanical features, and, therefore, on differentiating degrees of handedness. Alternative methodological approaches combined with TMS, such as high-density sEMG, might also contribute to discriminate handedness. Finally, the CSE might be a relevant measure to evaluate handedness while performing motor tasks but should be interpreted carefully due to the amount of variables involved.

## Author Contributions

MG, AAN-C, VM, and VS: conception, design, analysis, and interpretation of the literature. All authors have participated in drafting and critical revision of the manuscript for important intellectual content, final approval of the version to be submitted.

## Conflict of Interest

The authors declare that the research was conducted in the absence of any commercial or financial relationships that could be construed as a potential conflict of interest.
